# Evidence for the effectiveness of police-based pre-booking diversion programs in decriminalizing mental illness: A systematic literature review

**DOI:** 10.1371/journal.pone.0199368

**Published:** 2018-06-19

**Authors:** Carolyn S. Dewa, Desmond Loong, Austin Trujillo, Sarah Bonato

**Affiliations:** 1 Department of Psychiatry and Behavioral Sciences, University of California, Davis, Sacramento, California, United States of America; 2 Behavioral Health Center of Excellence, University of California, Davis, Sacramento, California, United States of America; 3 Institute for Mental Health Policy Research, Centre for Addiction and Mental Health, Toronto, Ontario, Canada; 4 Library Services, Centre for Addiction and Mental Health, Toronto, Ontario, Canada; Public Library of Science, UNITED KINGDOM

## Abstract

**Purpose:**

People with mental illnesses are at a significantly greater risk of police arrest than the general population. This pattern of arrests has been associated with a phenomenon referred to as the criminalization of mental illness such that people with mental illnesses are inappropriately diverted to the criminal justice system rather than to treatment. To decrease arrests of people with mental illnesses experiencing a crisis, pre-booking diversion programs have been developed to intervene at the point of police contact. This systematic literature review examines the state of knowledge regarding the effectiveness of police-based pre-booking diversion programs by addressing the question, “What is the evidence for the effectiveness of police-based pre-booking diversion programs in reducing arrests (i.e., reducing criminalization) of people with mental illnesses?”

**Methods:**

Systematic literature searches of seven databases were performed during May 2017. The searches sought to identify studies that examined the effectiveness of pre-booking diversion programs in decreasing arrests. A multi-phase screening process was completed independently by two pairs of reviewers as well as a risk of bias review.

**Results:**

A total of 2,750 unique citations were identified. Of these, 4 met the inclusion/exclusion criteria; all were from the US. Three of the studies examined the effectiveness of Crisis Intervention Teams and one study looked at a mobile crisis program. Two of the studies were at moderate risk of bias and two at high risk.

**Conclusions:**

This review indicates that this line of inquiry is still developing. There are a number of gaps yet to be filled. The current evidence for the effectiveness of police-based pre-booking diversion programs in reducing arrests (i.e., reducing criminalization) of people with mental illnesses is limited. However, these studies indicate there is moderate evidence that these programs increase linkages to mental health services.

## Introduction

The intersection between the justice and the healthcare systems ensnares a significant proportion of people with severe mental illness. Between 25–28% of people with mental illnesses have been arrested [1, 2]. Compared to people without mental illnesses, those with mental illnesses have significantly more police interactions [3]. For example, research from the US estimates that 7–10% of police contacts are with people with severe mental illnesses [4]. A Canadian study found that those with a mental illness were twice as likely to be arrested after controlling for the severity of the offence [5]. One of the primary consequences of these interactions is that people with mental illnesses are more likely to be arrested than the general population [6]. This pattern of arrests of people with mental illnesses has been associated with a phenomenon referred to as the criminalization of mental illness or the inappropriate diversion of people with mental illnesses to the criminal justice system rather than to treatment [7]. The criminalization has been attributed in part to barriers that police often encounter with the healthcare system such as long waiting periods in the emergency room and hospital admission refusals [8]. Because the criminal justice system appears to be more systematic and predictable than the healthcare system, police rely on it first rather than treatment [8].

Yet, treatment can be an alternative to arrests for people with mental illnesses. This is the case due to police discretion with regard to the outcomes of contacts [9]. For 12% of people with mental illnesses, police were involved in their pathway to service access [2]. But, for treatment rather than arrests to occur, it has been suggested that several conditions are required. These include increased training of police to handle crisis situations, increased coordination between police and mental health professionals, enhanced mental health services after arrest, and a move towards a treatment philosophy [8]. This translates into providing officers with supports to effectively deal with the often volatile crisis situations to which they are called [4]. It also requires linkages to mental health services that allow for direct referrals with no refusal policies [10]. In this way, arrests can decrease and the criminalization of mental illnesses can be addressed.

To decrease arrests of people with mental illnesses who are in crisis, criminal diversion programs have been developed to intervene at the point of police contact [10]. These programs are referred to as pre-booking or pre-charge diversion programs. They focus on police response and decision making; their goal is to redirect people with mental illnesses who have committed minor offences from arrest into mental health treatment [11].

Two primary types of police-based pre-booking responses have been described [12, 13]. One response uses a specialized police response in which specially trained officers respond to mental health crises and act as the link to mental health services [12]. Crisis Intervention Teams (CITs) are one of the most widely adopted types of specialized police response interventions [14, 15]. Another type of police-based response involves mental health professionals who respond to calls with officers as well as provide phone consultations to officers when questions arise in the field [12].

### Purpose of the systematic literature review

The purpose of this systematic literature review is to examine the state of knowledge regarding the effectiveness of police-based pre-booking diversion programs in addressing the criminalization of mental illnesses. We focus on police-based pre-booking diversion programs because the police are among the first to respond to a call [7]. Thus, police contact serves as the entry into the criminal justice system. One way to decrease entry is to intervene at the first contact point. Hence, we focused on police-based interventions.

We address the question, “What is the evidence for the effectiveness of police-based pre-booking diversion programs in reducing arrests (i.e., reducing criminalization) of people with mental illnesses?” As justice systems around the world seek effective interventions to increase treatment and decrease arrests [16], answers to this question may help inform decisions about adopting these interventions. The results of this review can also point to gaps in the literature and areas for future inquiry.

## Methods

This systematic review follows the *Preferred Reporting Items for Systematic Reviews and Meta-Analyses* (PRISMA) guidelines [17]. Because this review relies exclusively on published articles and did not involve primary data collection, approval from a Research Ethics Board was not required.

### Information sources

Seven electronic databases were searched:

1. *PsycINFO* (an index of journal articles, books, chapters, and dissertations in psychology, social sciences, behavioral sciences, and health sciences)
2. *Medline* (an index of biomedical research and clinical sciences journal articles)
3. *Medline In-Process* (an index of biomedical research and clinical sciences journal articles awaiting to be indexed into Medline)
4. *Embase* (an index of biomedical research, and abstracts from biomedical, drug and medical device conferences)
5. *Web of Science* (an index of journal articles, editorially selected books and conference proceedings in life sciences and biomedical research)
6. *Scopus* (an index of journal articles, books, and conference proceedings in science, technology, medicine, social science, and arts and humanities)
7. *Criminal Justice Abstracts* (an index of abstracts in criminal justice and criminology).

With a professional health science librarian (SB), search strategies were developed and tailored to each database and executed during May 2017 (S1 File). The Ovid platform was used to search *PsycINFO*, *Medline*, *Medline In-Process*, and *Embase*. *Web of Science* was searched using the Thomson Reuters search interface and *Scopus* was searched using the Elsevier platform. *Criminal Justice Abstracts* was searched using the EBSCO search platform. Across all databases, search results were limited to peer-reviewed articles published in English language journals but were not restricted by publication year.

### Study selection

To identify studies for inclusion, search results were screened using a three-stage screening process. Stage 1 involved screening article titles. Articles that passed Stage 1 were assessed for relevance based on their abstracts (Stage 2). In Stage 3, the remaining articles were screened based on their full text content. At the end of Stage 3, the reference lists of all articles that remained were hand searched. Articles that were identified through the hand search process were subjected to the three-stage screening process.

The entire multi-phase screening process was completed independently by two pairs of reviewers (CSD and AT, CSD and DL). The inter-rater reliability corrected for chance [18] between CSD & AT and CSD & DL was κ = 0.78 and κ = 0.96, respectively. Where there were inconsistent ratings between the two reviewers, the discrepancies were discussed until consensus was reached.

### Eligibility criteria

For the purposes of this review, we include police-based pre-booking diversion programs defined by the elements described by Borum and Franz [11] such that the intervention: (1) focuses on police response and decision making, (2) has the goal of diverting people with mental illnesses who have committed minor offences from arrest into mental health treatment, and (3) involves a collaboration with mental health treatment providers.

To identify relevant studies, the following eligibility criteria were used:

1. The study reports on a police-based pre-booking diversion intervention
2. The study’s intervention involves a collaboration with the police and mental health providers
3. The study’s intervention is initiated by police contact
4. The study’s intervention responds to a crisis or emergency involving a person with a mental illness (including people with concurrent disorders)
5. The study’s intervention provides services to address crises (i.e., it is time limited)
6. The study outcomes involve arrests
7. The study reports on adults (18 years or older) with mental illnesses who are in the community (including people with concurrent disorders)
8. The study is original research
9. The study uses a comparable comparison group for usual practice

Studies were excluded if they looked:

1. Exclusively at people with substance use disorders
2. Exclusively at people who were violent

### Risk of bias assessment

A 7-item checklist was used to assess the risk of bias of the included studies. This checklist was adapted from *Cochrane Handbook for Systematic Reviews of Interventions* [19] and Dewa et al.’s [20] Risk of Bias Assessment Tool. It considers the following:

1. Adequate sequence generation (group assignments of participants are based on chance)
2. Allocation concealment (schedule of random assignments are kept concealed from staff involved in study enrollment)
3. Blinding (participants and study staff are masked of the knowledge of which intervention was received)
4. Incomplete outcome data (there is no significant difference between groups who withdraw from the study)
5. Selective reporting (based on the study outcomes, all study outcomes are complete and not selectively reported)
6. Intervention adherence (a process is in place to ensure fidelity to the intervention model)
7. Recruitment strategy (recruitment process is open to all potential participants who meet the study eligibility criteria)

There were three possible ratings for each criterion: -1 (if there was a high risk of bias), +1 (if there was a low risk of bias) or 0 (if there was insufficient information to assess risk). Steps were not taken to contact the authors of studies to gather additional information or potential related information. Articles with a calculated score of less than 50% were considered to have a high risk of bias. Scores between 50% and 79% were considered to have moderate risk. Studies with a score of 80% or greater were considered to have low risk of bias.

The risk of bias assessment results were also used as part of the criteria to conduct a meta-analysis. To be included in the meta-analysis, a study had to have been assessed as either having a low or moderate risk of bias. Two of the four studies met this criterion. A second criterion was that the models that were being studied needed to be similar. There were significant differences in the designs of the models examined in the two remaining studies. One was an intensive training model to equip officers in the field. The other was based on consultations provided to officers from mental health specialists. Thus, the decision was made to forgo conducting a meta-analysis.

### Data abstraction

Two reviewers (CSD and DL) abstracted the data from each of the articles by filling in table shells containing headings. The study description data included: (1) Type of experimental intervention and the comparison intervention, (2) Numbers of participants in each study arm, (3) Study period, (4) Type of study design used, (5) Number and timing of data collection points, and (6) Types of outcomes measured. The study results data that were collected included: (1) Statistical parameter calculated and the values as well as the measure of uncertainty if indicated. Disagreements were discussed until consensus was reached.

## Results

### Article inclusion and exclusion results

**Fig 1:**
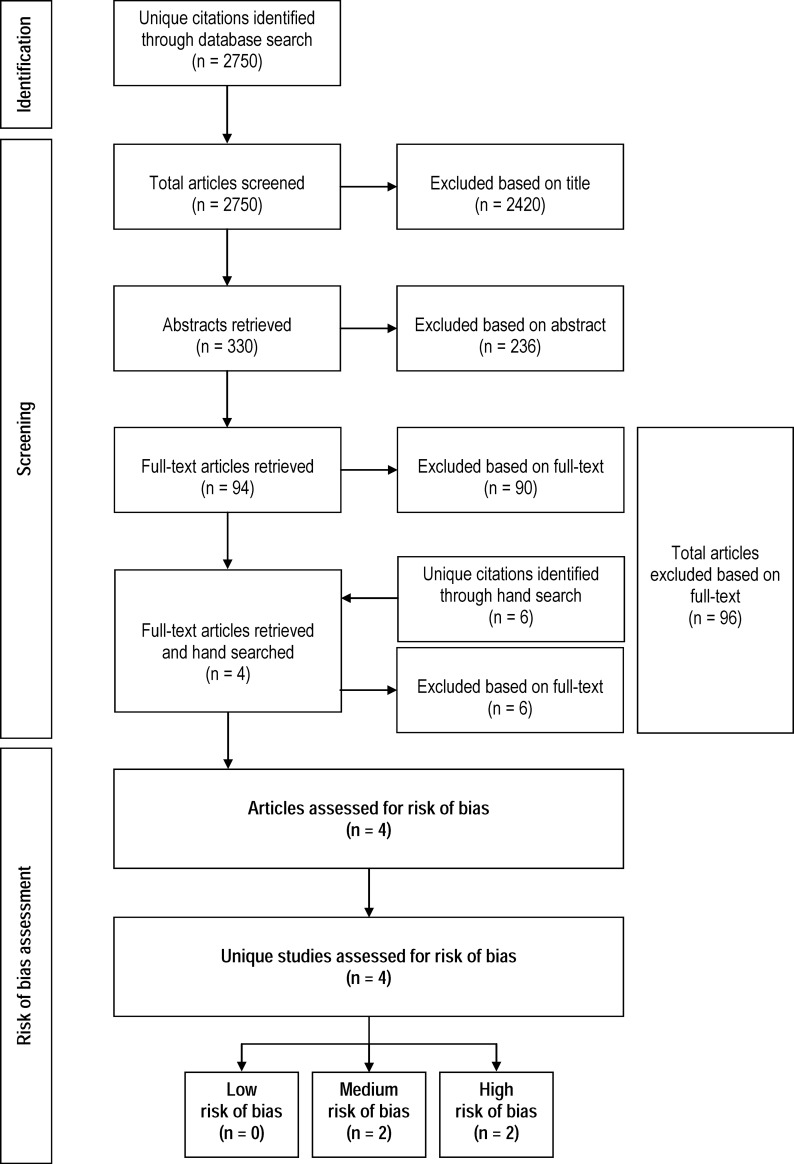
Flowchart of accepted and rejected articles.

The electronic literature search resulted in the identification of 2,750 unique citations (Fig 1). At the end of the title review, 2,420 citations were excluded; this left 330 articles for abstract review. During the abstract review, another 236 citations were excluded; this left 94 articles for full-text review. Reasons for article exclusions at full text review were: (1) not a pre-booking diversion program (n = 22), (2) the study did not examine relevant outcome (n = 15), (3) it was not original research (n = 40) and (4) no comparison group (n = 12). After the full-text review, 4 articles remained and their reference lists were hand searched for relevant studies. The hand search identified six additional citations. But, all were excluded at full text review.

### Risk of bias assessment results

**Fig 2:**
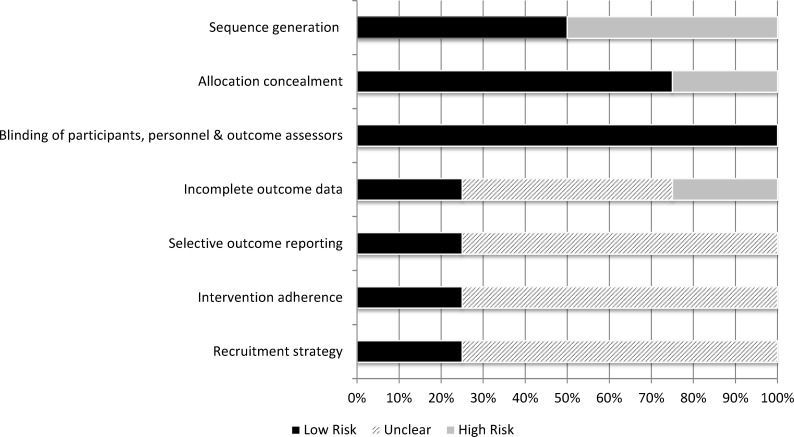
Overview of potential bias across studies.

In terms of potential risk of bias, our assessment identified 2 of the 4 studies to have a high risk of bias and 2 to have moderate risk. Fig 2 shows the potential bias for these studies. None of the studies were randomized controlled trials; all used quasi-experimental designs (S2 File). Because of the nature of the intervention, none of them could conceal allocation. The studies offered few details regarding either the characteristics of the sample participants compared to the group from which they were drawn or the missing data. The studies did not indicate whether there was a check for intervention adherence during the study.

### Overview of the studies

There were four studies that met the inclusion criteria (Table 1). All of the studies were from the US; two from Georgia, one from Ohio, and one from Illinois.

**Table 1:** Summary of studies.

Author(s)	Intervention(s)	Study Population	Study Design Data Points	Relevant Outcomes
Compton et al. [21] (Four counties in Georgia, USA)	• Intervention: Crisis Intervention Team (CIT). Officers receive 40-hours of training to identify signs and symptoms of mental illness, to de-escalate crisis situations, and make appropriate dispositions. Collaboration with mental health community • Comparison: Non-CIT trained officers	• n = 180 officers (91 with CIT training and 89 without training) • n = 1,063 police encounters with people with serious mental illness, alcohol or drug problem or developmental disability • Study period: Mar–Nov 2010	• Study Design: Prospective officer self-report • Data points: 30 encounters/officer with people with serious mental illness, alcohol or drug problem or developmental disability during a 6-week period	1. Encounter disposition: arrest; referral; resolution
Scott [22] (Georgia, USA)	• Intervention: Mobile crisis program to provide community-based services to stabilize people in psychiatric crisis in the least restrictive environment, to decrease arrests of people with mental illnesses and to decrease officer time for each of these calls. Team provides consultation to officers by phone or radio or by assisting officers in the field. • Comparison: 911 calls identified as psychiatric emergencies handled by officers without mobile crisis team help	• n = 131 psychiatric emergencies (73 with the team and 58 without) • Study period: Oct–Dec 1995	• Study Design: Retrospective administrative data	1. Arrests 2. Hospitalizations
Teller et al. [23] (Ohio, USA)	• Intervention: CIT with modifications from the Memphis model. One modification, for people with comorbid non-psychiatric conditions, was referral to a general hospital emergency department instead of or before going to psychiatric emergency services. The second modification was inclusion of paramedics in CIT training. • Comparison: Pre-CIT training and Post-CIT trained vs. non-trained	• n = 4,367 calls related to mental disturbances • Study period: May 1998 –Apr 2004	• Study Design: Pre/Post CIT training and in Post CIT, CIT vs. non-trained using administrative data • Pre: Two years • Post: Four years	1. Call disposition: transport to psychiatric emergency; transport to other treatment facility; jail; no need for transport
Watson et al. [9] (Illinois, USA)	• Intervention: CIT adapted modification of the Memphis model. 40-hour modular curriculum covering signs and symptoms, risk assessment/intervention, and role-play to enhance de-escalation skills. Special focus was on sub-populations: child and adolescent, substance abuse, and geriatric. • Comparison: Early stages of CIT implementation	• n = 4 police districts (2 districts with CIT experience and 2 districts in which CIT was newly introduced) • Used proportionate random sampling to select officers to invite • n = 112 officer participants (56 experienced with CIT and 56 recently trained-CIT) • Study period: Mar–Apr 2008	• Study Design: Officer interviews asked about experiences over past month	1. Call disposition: Without arrest, directed to mental health/social services; arrest; contact only

### Description of the study populations

All four of the studies were limited to selected policing districts. In two of the studies, officers were recruited to participate [9, 21]. In Compton et al.’s [21] study, officers were asked to record information about their 30 encounters with people with serious mental illness, alcohol or drug problem, or developmental disability during a 6-week period. Identification of people with the conditions of interest was dependent on the officer. In contrast, Watson et al. [9] interviewed officers about their experiences over the past month.

Two studies depended on retrospective administrative data; these data included dispatch records, police notes, public safety records, and intervention program records [22, 23]. Teller et al. [23] looked at mental disturbance calls that were identified by police dispatchers. Scott [22] focused on calls related to psychiatric emergencies; it was not clear how the psychiatric emergencies were identified–by the caller, by dispatch, or by the officer.

### Interventions and comparison groups

Two primary types of interventions were examined: CIT [9, 21, 23] and a mobile crisis program [22]. The studies that included CIT programs were based on the Memphis model [15] offering 40 hours of training that focus on officer and consumer safety, proper referral to those in crisis, and de-escalation skills training [15]. Two of the CIT interventions studied noted modifications to training to reflect the context in which the training was being conducted [9, 23]. The comparisons for these CIT programs were either the outcomes related to pre-intervention [23] and/or groups who had not received CIT training [9, 21, 23].

In Scott’s [22] study, the mobile crisis program was designed to respond to 911 psychiatric emergency calls with the goal of stabilizing people in psychiatric crisis in the least restrictive environment possible. The team was comprised of two police officers and one psychiatric nurse available seven days a week from 3 p.m. to 10:30 p.m. Because there was only one team and it was not available 24-hours a day, there were some 911 psychiatric emergency calls to which the mobile crisis program did not respond. These calls formed the comparison group for the mobile crisis team intervention group.

### Outcomes

Three of the four studies that looked at the differences in arrest rates between the intervention and comparison groups did not find significantly different results [9, 22, 23] (Table 2). However, Compton et al.’s [21] study of CIT did report significant differences in favor of the CIT group. In addition, all four studies consistently reported significant differences in the rate of referrals to mental health services of the intervention versus comparison group. Referral was more likely in the intervention groups [9, 21–23].

**Table 2:** Summary of study call disposition outcomes.

Author(s)	Intervention(s)	Arrest	Referral	Resolution
Compton et al. [21] (Georgia, USA)	• Intervention: Crisis Intervention Team (CIT). Officers receive 40-hours of training to identify signs and symptoms of mental illness, to deescalate crisis situations, and make appropriate dispositions. Collaboration with mental health community • Comparison: Non-CIT trained officers	• CIT: 13% • Non-CIT: 24% • Odds ratio = 0.47, p < 0.01	• CIT: 40% • Non-CIT: 29% • Odds ratio = 1.70, p = 0.03	• CIT: 47% • Non-CIT: 48% • Odds ratio = 0.96, p = 0.85
Scott [22] (Georgia, USA)	• Intervention: Mobile crisis program to provide community-based services to stabilize people in psychiatric crisis in the least restrictive environment, to decrease arrests of people with mental illnesses and to decrease officer time for each of these calls. Team provides consultation to officers by phone or radio or by assisting officers in the field. • Comparison: 911 calls identified as psychiatric emergencies handled by officers without mobile crisis team help	• Mobile crisis: 7% •No mobile crisis: 14% •Difference not significant	Any psychiatric hospitalization: • Mobile crisis: 45% • No mobile crisis: 72% • (X^2^ (1) = 8.24, p < 0.01) Voluntary hospitalization: • Mobile crisis: 64% • No mobile crisis: 33% • X^2^ (1) = 13.9, p < 0.001	
Teller et al. [23] (Ohio, USA)	• Intervention: CIT with modifications from the Memphis model. One modification, for people with comorbid non-psychiatric conditions, was referral to a general hospital emergency department instead of or before going to psychiatric emergency services. The second modification was inclusion of paramedics in CIT training. • Comparison: Pre-CIT training and Post-CIT implementation CIT trained vs. non-trained	Transport to jail: • Pre-CIT: 6.5% • Post-CIT: 6.0% • CIT trained officers: 7.0% • Non-CIT trained officers: 5.2% • No significant differences between Pre-CIT vs Post-CIT and CIT trained vs non-CIT	Transport to psychiatric emergency services: • Pre-CIT: 25.8% • Post-CIT: 28.0% • CIT trained officers: 32.8% • Non-CIT trained officers: 26.6% • Significant differences for CIT- trained vs pre-CIT and CIT-trained vs Non-CIT, p = 0.001	• Pre-CIT: 54.3% • Post-CIT: 52.4% • CIT trained officers: 44.3% • Non-CIT trained officers: 55.9% • Significant differences for CIT-trained vs Pre-CIT and CIT-trained vs Non-CIT, p < 0.001
Watson et al. [9] (Illinois, USA)	• Intervention: CIT adapted modification of the Memphis model. 40-hour modular curriculum covering signs and symptoms, risk assessment/intervention, and role-play to enhance de-escalation skills. Special focus was on sub-populations: child and adolescent, substance abuse, and geriatric. • Comparison: Early stages of CIT implementation	Proportion of people arrested: • CIT vs non-CIT: β = -0.06 • Difference not significant	Proportion of people directed to mental health: • CIT vs non-CIT: β = 0.17 • p < 0.05	Proportion of people with contact only: • CIT vs non-CIT: β = -0.09 • Difference not significant

## Discussion

Our systematic literature review identified four studies examining the effectiveness of police-based pre-booking diversion programs compared to standard practice. Two of the studies were at moderate risk of bias and two at high risk. These results suggest that one of the main challenges with this type of research is that it is difficult to avoid the risk of bias. The police-based pre-booking interventions involve either police training or the addition of a mental health team. It is difficult to blind study participants to either. It also seems to be difficult to identify objective sources of information about psychiatric crises and factors associated with the decision process regarding arrests, release, and treatment. This can be attributed to the fact that because it is police-based, information is not necessarily collected about a suspect’s health status or other factors that are not associated with law enforcement. This creates difficulties when trying to identify a comparison group and information about the comparison.

Among the included studies, there were two intervention models tested: CIT and mobile crisis. Both types of interventions have the objective of appropriately diverting people with mental illnesses from the criminal justice system into mental health services. The results of the studies suggest that compared to usual practice, CIT and mobile crisis can significantly increase the probability of people accessing mental health services. However, the effects of these programs on arrest rates are equivocal.

These results suggest that these pre-booking police-based diversion interventions achieve part of their objective; they divert people into care. But, effectiveness in linkages to service seems to be related to a variety of moderating factors. For example, Watson et al. [24] observed that the degree of availability of mental health services is positively associated with diversion into care. They conclude that in communities where mental health resources are accessible, CIT trained officers are more likely to link people to mental health services than are officers who have not been trained. However, service availability was not associated with arrests [24].

Part of reason that the availability of mental health services does not appear to prevent arrests [24] may be related to the nature of reason for the contact. Ritter et al. [25] report that people suspected of illicit drug use or of violence to others are more likely to be taken to jail than to treatment; these two call types account for 12.8% and 18.5% of total police contacts with people with mental illnesses, respectively. This suggests that among people with mental illnesses, there is a group of people demonstrating behaviors that result in arrest. One way to address this could be by broadening the scope of pre-booking intervention to allow for earlier intervention to prevent police contact. As with CIT and mobile crisis programs, earlier intervention also involves mental health services. For example, Earl et al. [26] describe a program called the Neighbourhood Outreach Scheme (NOS) that was designed to pre-empt crises and police contact. In this program, a community psychiatric nurse accepts referrals from police and mental health specialists to follow-up with vulnerable people from the neighborhood who do not meet thresholds for a mental health crisis or criminal intervention. They found a significant decrease in the number of police contacts at 6-month follow-up. This suggests a role for diversion that seeks to prevent the need for police contact by intervening further upstream but still involving a partnership between the police and mental health services.

### Future directions

In addition to developing study designs that decrease the risk of bias, it would also be important to understand the barriers to implementing these types of interventions. For example, CIT requires 40 hours of training. That may be too resource intensive for some jurisdictions. If this is the case, it may be important to understand the critical ingredients of the police-based pre-booking programs. In this way, if a region is not able to offer 40 hours of training to all officers, it might be possible to abbreviate the training. In other words, what parts should not be eliminated? In the case of the mobile crisis program, what are the most essential hours for their availability? Are there different types of mental health providers that should be available at the different hours?

In addition, Steadman and Morrissette [27] point out that for these types of programs to be effective, there needs to be mental health community resources to which police can refer people. Furthermore, police should be considered one part of the crisis care continuum. In other words, the context into which these types of programs are introduced are critical. Future research could help in understanding about how to implement these types of programs in environments where resources are scarce.

Finally, there is evidence that training can increase knowledge, decrease negative attitudes towards mental illness, and improve assessment strategies [28]. But, there is less information about whether implicit stigma is affected [28]. This could be important to understand if this is one of factors in the pathways that lead to arrests. If this is the case, implicit stigma could also be a barrier to the effectiveness of these interventions.

### Strengths and limitations of the search strategy

Although seven databases were used in the search, articles that did not appear in any of the databases would not have been included in this review. To ensure that we used a sufficiently broad search strategy, we employed broad search terms for each database and hand searched relevant articles.

Another limitation is related to the fact that the search was limited to articles published in English-language journals. Studies that were not published in English would have been excluded and this review and their evidence would not have been considered. Furthermore, the focus on published peer-reviewed also introduced a potential limitation. Our results may be subject to publication bias [29]. That is, there is evidence that non-results are often not published [30]. As such, studies that did not have significant results would not have been included. This means that there may be a group of unpublished studies that indicate certain interventions are not effective. Addressing this would necessitate including gray literature. However, it should also be noted that the quality of the gray literature has been questioned because it is not necessarily subject to critical assessment before it is publically released [31]. Consequently, studies that have not been published may be at risk of being of lower quality.

## Conclusions

The evidence on the effectiveness of police-based pre-booking diversion programs in reducing arrests (i.e., reducing criminalization) of people with mental illnesses is limited. In addition, the existing evidence is not strong. There is moderate evidence that these programs increase linkages to mental health services. These results suggest the need for more studies that incorporate research designs that decrease the risk of bias. Preliminary findings also suggest that the contexts in which the intervention studies are conducted are also an important consideration. In the same way that these interventions require collaboration between criminal justice and mental health, addressing the limitations in this literature will require collaborations among criminal justice, mental health and research.

### Compliance with ethical standards

#### Research involving human participants

All procedures performed in the study involving human participants were in accordance with the ethical standards of the institutional research committee and with the 1964 Helsinki declaration and its later amendments or comparable ethical standards.

#### Informed consent

All data for this study were gathered from publicly available published articles and did not involve primary data collection. Therefore, informed consent was not gathered.

## Supporting information

S1 File Search terms and keywords. (PDF)

S2 File Risk assessment of bias checklist. (PDF)

S3 File PRISMA checklist. (PDF)
