# Supplementary material for: Evidence for the effectiveness of police-based pre-booking diversion programs in decriminalizing mental illness: A systematic literature review
Source: PLoS One. 2018 Jun 19;13(6):e0199368. doi: 10.1371/journal.pone.0199368 (PMC6007921; doi:10.1371/journal.pone.0199368)
Supplement: S1 File — (PDF) [file pone.0199368.s001.pdf]

Search Strategy

Database**:** PsycINFO

Search Terms**:** [exp Mental Disorders/ OR (mental$ adj3 disorder$).mp. OR exp Psychiatric patients/ OR (mental$ adj3 ill$).mp. OR (psychiatric$ adj3 disorder$).mp. OR (psychiatric$ adj3 ill$).mp. OR exp Mentally Ill Offenders/ OR Psychiatry/ OR psychiatr$.mp. OR exp Community Psychiatry/ OR Forensic Psychiatry/ OR (forens$ adj3 jurisprud$).mp. OR exp "Commitment (Psychiatric)"/ OR (outpatient$ adj3 commit$).mp. OR (involuntar$ adj3 commit$).mp. OR (commit$ adj3 mental$ adj3 ill$).mp. OR (commit$ adj3 voluntar$).mp. OR (duration$ adj3 commit$).mp. OR exp Community Mental Health Centers/ OR (communit$ adj3 mental$ adj3 health$ adj3 cent$).mp. OR cmhc.mp.] **AND** [(mental$ adj3 health$ adj3 justice$).mp. OR (mental$ adj3 ill$ adj3 justice$).mp. OR diversion$.mp. OR divert$.mp. OR exp legal arrest/ OR arrest$.mp. OR exp Crisis Intervention/ OR exp Crisis Intervention Services/ OR exp Intervention/ OR interven$.mp.] **AND** [exp Police Personnel/ OR exp Law Enforcement/ OR police$.mp. OR law$ enforcement$.mp. OR (mobile$ adj3 health$ adj3 unit$).mp. OR (mobile$ adj3 clinic$).mp.]

Database: Medline

Search Terms: [exp Mental Disorders/ OR (mental$ adj3 disorder$).mp. OR exp Mentally Ill Persons/ OR (mental$ adj3 ill$).mp. OR (psychiatric$ adj3 disorder$).mp. OR (psychiatric$ adj3 ill$).mp. OR Psychiatry/ OR psychiatr$.mp. OR exp Community Psychiatry/ OR Forensic Psychiatry/ OR (forens$ adj3 jurisprud$).mp. OR exp

"Commitment of Mentally Ill"/ OR (outpatient$ adj3 commit$).mp. OR (involuntar$ adj3 commit$).mp. OR (commit$ adj3 mental$ adj3 ill$).mp. OR (commit$ adj3 voluntar$).mp. OR (duration$ adj3 commit$).mp. OR exp Community Mental Health Centers/ OR (communit$ adj3 mental$ adj3 health$ adj3 cent$).mp. OR cmhc.mp.] **AND** [(mental$ adj3 health$ adj3 justice$).mp. OR (mental$ adj3 ill$ adj3 justice$).mp. OR diversion$.mp. OR divert$.mp. OR arrest$.mp. OR exp Crisis Intervention/ OR exp Intervention Studies/ OR interven$.mp.] **AND** [exp Police/ OR exp Law Enforcement/ OR police$.mp. OR law$ enforcement$.mp. OR (mobile$ adj3 health$ adj3 unit$).mp. OR (mobile$ adj3 clinic$).mp.]

Database: Medline In-Process

Search Terms: [exp Mental Disorders/ OR (mental$ adj3 disorder$).mp. OR exp Mentally Ill Persons/ OR (mental$ adj3 ill$).mp. OR (psychiatric$ adj3 disorder$).mp. OR (psychiatric$ adj3 ill$).mp. OR Psychiatry/ OR psychiatr$.mp. OR exp Community Psychiatry/ OR Forensic Psychiatry/ OR (forens$ adj3 jurisprud$).mp. OR exp "Commitment of Mentally Ill"/ OR (outpatient$ adj3 commit$).mp. OR (involuntar$ adj3 commit$).mp. OR (commit$ adj3 mental$ adj3 ill$).mp. OR (commit$ adj3 voluntar$).mp. OR (duration$ adj3 commit$).mp. OR exp Community Mental Health Centers/ OR (communit$ adj3 mental$ adj3 health$ adj3 cent$).mp. OR cmhc.mp.] **AND** [(mental$ adj3 health$ adj3 justice$).mp. OR (mental$ adj3 ill$ adj3 justice$).mp. OR diversion$.mp. OR divert$.mp. OR arrest$.mp. OR exp Crisis Intervention/ OR exp Intervention Studies/ OR interven$.mp.] **AND** [exp Police/ OR exp Law Enforcement/ OR police$.mp. OR law$ enforcement$.mp. OR (mobile$ adj3 health$ adj3 unit$).mp. OR (mobile$ adj3 clinic$).mp.]

Database: Embase

Search Terms: [exp Mental Disease/ OR (mental$ adj3 disorder$).mp. OR exp Mental Patient/ OR (mental$ adj3 ill$).mp. OR (psychiatric$ adj3 disorder$).mp. OR (psychiatric$ adj3 ill$).mp. OR Psychiatry/ OR psychiatr$.mp. OR exp Social Psychiatry/ OR Forensic Psychiatry/ OR (forens$ adj3 jurisprud$).mp. OR exp Involuntary Commitment/ OR (outpatient$ adj3 commit$).mp. OR (involuntar$ adj3 commit$).mp. OR (commit$ adj3 mental$ adj3 ill$).mp. OR (commit$ adj3 voluntar$).mp. OR (duration$ adj3 commit$).mp. OR exp Community Mental Health Centers/ OR (communit$ adj3 mental$ adj3 health$ adj3 cent$).mp. OR cmhc.mp.] **AND** [(mental$ adj3 health$ adj3 justice$).mp. OR (mental$ adj3 ill$ adj3 justice$).mp. OR diversion$.mp. OR divert$.mp. OR arrest$.mp. OR exp Crisis Intervention/ OR exp Intervention Study/ OR interven$.mp.] **AND** [exp Police/ OR exp Law Enforcement/ OR police$.mp. OR law$ enforcement$.mp. OR (mobile$ adj3 health$ adj3 unit$).mp. OR (mobile$ adj3 clinic$).mp.]

Database: Web of Science

Search Terms: [mental* NEAR/3 disorder* OR mental* NEAR/3 ill* OR psychiatric* NEAR/3 disorder* OR psychiatric* NEAR/3 ill* OR psychiatr* OR forens* NEAR/3 jurisprud* OR outpatient* NEAR/3 commit* OR involuntar* NEAR/3 commit* OR commit* NEAR/3 mental* NEAR/3 ill* OR commit* NEAR/3 voluntar* OR duration* NEAR/3 commit* OR communit* NEAR/3 mental* NEAR/3 health* NEAR/3 cent* OR

cmhc] **AND** [mental* NEAR/3 health* NEAR/3 justice* OR mental* NEAR/3 ill* NEAR/3 justice* OR diversion* OR divert* OR arrest* OR interven*] **AND** [police* OR law* enforcement* OR mobile* NEAR/3 health* NEAR/3 unit* OR mobile* NEAR/3 clinic*]

<u>Database</u>**:** Criminal Justice Abstracts

<u>Search Terms</u>**:** [mental* NEAR/3 disorder* OR mental* NEAR/3 ill* OR psychiatric* NEAR/3 disorder* OR psychiatric* NEAR/3 ill* OR psychiatr* OR forens* NEAR/3 jurisprud* OR outpatient* NEAR/3 commit* OR involuntar* NEAR/3 commit* OR commit* NEAR/3 mental* NEAR/3 ill* OR commit* NEAR/3 voluntar* OR duration* NEAR/3 commit* OR communit* NEAR/3 mental* NEAR/3 health* NEAR/3 cent* OR cmhc] **AND** [mental* NEAR/3 health* NEAR/3 justice* OR mental* NEAR/3 ill* NEAR/3 justice* OR diversion* OR divert* OR arrest* OR interven*] **AND** [police* OR law* enforcement* OR mobile* NEAR/3 health* NEAR/3 unit* OR mobile* NEAR/3 clinic*]

<u>Database</u>**:** Scopus

<u>Search Terms</u>**:** [mental* W/3 disorder* OR mental* W/3 ill* OR psychiatric* W/3 disorder* OR psychiatric* W/3 ill* OR psychiatr* OR forens* W/3 jurisprud* OR outpatient* W/3 commit* OR involuntar* W/3 commit* OR commit* W/3 mental* W/3 ill* OR commit* W/3 voluntar* OR duration* W/3 commit* OR communit* W/3 mental* W/3 health* W/3 cent* OR cmhc] **AND** [mental* W/3 health* W/3 justice* OR mental* W/3 ill* W/3 justice* OR diversion* OR divert* OR arrest* OR interven*] **AND** [police* OR law* enforcement* OR mobile* W/3 health* W/3 unit* OR mobile* W/3 clinic*]
