# Supplementary material for: Evidence for the effectiveness of police-based pre-booking diversion programs in decriminalizing mental illness: A systematic literature review
Source: PLoS One. 2018 Jun 19;13(6):e0199368. doi: 10.1371/journal.pone.0199368 (PMC6007921; doi:10.1371/journal.pone.0199368)
Supplement: S2 File — (PDF) [file pone.0199368.s002.pdf]

Risk of Bias Assessment Checklist

Author(s)	1	2	3	4	5	6	7	Total Score	Total Score Percentage
Compton et al. [21]	-1	-1	1	-1	0	0	0	-2	-28.57%
Scott [22]	1	1	1	0	0	1	0	4	57.14%
Teller et al. [23]	-1	1	1	1	1	0	0	3	42.86%
Watson et al. [9]	1	1	1	0	0	0	1	4	57.14%

Risk of Bias Assessment Criteria

1. Adequate sequence generation (group assignments of participants are based on chance)
2. Allocation concealment (schedule of random assignments are kept concealed from staff involved in study enrollment)
3. Blinding (participants and study staff are masked of the knowledge of which intervention was received)
4. Incomplete outcome data (there is no significant difference between groups who withdraw from the study)
5. Selective reporting (based on the study outcomes, all study outcomes are complete and not selectively reported)
6. Intervention adherence (a process is in place to ensure fidelity to the intervention model)
7. Recruitment strategy (recruitment process is open to all potential participants who meet the study eligibility criteria)
